# Efficacy and durability of multifactorial intervention on mortality and MACEs: a randomized clinical trial in type-2 diabetic kidney disease

**DOI:** 10.1186/s12933-021-01343-1

**Published:** 2021-07-16

**Authors:** Ferdinando Carlo Sasso, Pia Clara Pafundi, Vittorio Simeon, Luca De Nicola, Paolo Chiodini, Raffaele Galiero, Luca Rinaldi, Riccardo Nevola, Teresa Salvatore, Celestino Sardu, Raffaele Marfella, Luigi Elio Adinolfi, Roberto Minutolo, NID-2 Study Group Investigators, U. Amelia, C. Acierno, P. Calatola, O. Carbonara, A. Caturano, G. Conte, G. Corigliano, M. Corigliano, R. D’Urso, A. De Matteo, L. De Nicola, N. De Rosa, E. Del Vecchio, G. Di Giovanni, A. Gatti, S. Gentile, L. Gesuè, L. Improta, A. Lampitella, A. Lampitella, A. Lanzilli, N. Lascar, S. Masi, P. Mattei, V. Mastrilli, P. Memoli, R. Minutolo, R. Nasti, A. Pagano, M. Pentangelo, E. Pisa, E. Rossi, F. C. Sasso, S. Sorrentino, R. Torella, R. Troise, P. Trucillo, A. A. Turco, S. Turco, F. Zibella, L. Zirpoli

**Affiliations:** 1 Department of Advanced Medical and Surgical Sciences, University of Campania “Luigi Vanvitelli”, Piazza Luigi Miraglia 2, 80138 Naples, Italy; 2 Medical Statistics Unit, Department of Physical and Mental Health and Preventive Medicine, University of Campania “Luigi Vanvitelli”, Piazza Luigi Miraglia 2, 80138 Naples, Italy; 3 Department of Precision Medicine, University of Campania “Luigi Vanvitelli”, Via De Crecchio 7, 80138 Naples, Italy

**Keywords:** Diabetic nephropathy, Multifactorial intervention, Intensified treatment, CV risk factors, Very high CV risk, MACE

## Abstract

**Background:**

Multiple modifiable risk factors for late complications in patients with diabetic kidney disease (DKD), including hyperglycemia, hypertension and dyslipidemia, increase the risk of a poor outcome. DKD is associated with a very high cardiovascular risk, which requires simultaneous treatment of these risk factors by implementing an intensified multifactorial treatment approach. However, the efficacy of a multifactorial intervention on major fatal/non-fatal cardiovascular events (MACEs) in DKD patients has been poorly investigated.

**Methods:**

Nephropathy in Diabetes type 2 (NID-2) study is a multicentre, cluster-randomized, open-label clinical trial enrolling 395 DKD patients with albuminuria, diabetic retinopathy (DR) and negative history of CV events in 14 Italian diabetology clinics. Centres were randomly assigned to either Standard-of-Care (SoC) (n = 188) or multifactorial intensive therapy (MT, n = 207) of main cardiovascular risk factors (blood pressure < 130/80 mmHg, glycated haemoglobin < 7%, LDL, HDL and total cholesterol < 100 mg/dL, > 40/50 mg/dL for men/women and < 175 mg/dL, respectively). Primary endpoint was MACEs occurrence by end of follow-up phase. Secondary endpoints included single components of primary endpoint and all-cause death.

**Results:**

At the end of intervention period (median 3.84 and 3.40 years in MT and SoC group, respectively), targets achievement was significantly higher in MT. During 13.0 years (IQR 12.4–13.3) of follow-up, 262 MACEs were recorded (116 in MT vs. 146 in SoC). The adjusted Cox shared-frailty model demonstrated 53% lower risk of MACEs in MT arm (adjusted HR 0.47, 95%CI 0.30–0.74, *P* = *0.001*). Similarly, all-cause death risk was 47% lower (adjusted HR 0.53, 95%CI 0.29–0.93, *P* = *0.027*).

**Conclusion:**

MT induces a remarkable benefit on the risk of MACEs and mortality in high-risk DKD patients.

*Clinical Trial Registration* ClinicalTrials.gov number, NCT00535925. https://clinicaltrials.gov/ct2/show/NCT00535925

**Supplementary Information:**

The online version contains supplementary material available at 10.1186/s12933-021-01343-1.

## Background

Type 2 diabetes mellitus (T2DM) patients are at high risk of death, myocardial infarction (MI) and stroke compared to the general population [1]. More important, onset of diabetic kidney disease (DKD) remarkably worsens cardiovascular prognosis, as demonstrated by a very large meta-analysis showing that hazards for cardiovascular mortality at a given eGFR/albuminuria are higher among T2DM patients throughout the whole spectrum of disease [2]. The major role of DKD on cardiovascular outcome has also been supported by an observational study on 1.3 million of patients reporting a higher incidence rate of MI in DKD patients versus diabetes or chronic kidney disease alone [3]. Of note, this finding is enhanced by the coexistence of proteinuria; indeed, albuminuria and low eGFR per se accelerate atherosclerosis process. Such a marked cardiovascular risk significantly modifies the outcome of DKD patients who often do not survive long enough to reach the natural fate of end-stage kidney disease [3–6].

The importance of a multifactorial approach in T2DM has been emphasized by the analysis of Swedish National Diabetes Register comparing prognosis of ~ 270,000 T2DM patients versus ~ 1,300,000 age- and gender-matched controls [1]. In particular, T2DM patients with five risk-factors within target range showed either a small or any excess risk of death, MI or stroke, as compared with controls [1]. However, DKD was either mild or absent in the vast majority of patients (mean GFR 84 mL/min and 4.9% with macroalbuminuria). Furthermore, achievement of targets for multiple risk factors was uncommon (5%).

As for observational studies, also multi-target interventional trials in DKD are lacking. The Steno-2 is the only study evaluating the effects of multifactorial approach in 160 microalbuminuric patients. The trial disclosed a significant reduction of both mortality and cardiovascular risk after implementing an intensified approach to multiple targets [7, 8]. However, Steno-2 was a single-centre study, carried out in patients with preserved renal function (mean GFR 118 mL/min) and mostly without diabetic retinopathy (DR) (74%), that is, a sign of microangiopathy strictly associated with cardiovascular risk besides being a recognized marker of DKD [9].

Aim of the present trial is to evaluate the efficacy of a multifactorial intensive therapy (MT) versus Standard-of-Care (SoC) on major fatal and non-fatal cardiovascular events (MACEs) in a population with DKD patients with albuminuria and retinopathy. Durability of the effect of intensified treatment was tested by extending follow-up to several years after the end of intervention phase.

## Methods

### Trial design

The Nephropathy In Diabetes type 2 (NID-2) study is an open-label cluster randomized clinical trial in a population referred to 14 Italian diabetology clinics [10]. To maximize the contrast between the two approaches, we randomized clinics rather than patients. Indeed, in the latter modality of randomization similarities between the two interventions are expected to ensue over the long-term. Centres were randomly assigned to either MT therapy or SoC. A questionnaire ascertained that all participating physicians were well aware of the guidelines on T2DM management published at the time of the study [11–14].

All MACEs diagnoses were performed in each patient according to the diagnostic criteria defined by the international standards of care guidelines [15–17]. MACEs were evaluated by cardiologist blinded to the study arm (MT or SoC), either belonging to the same Centres or to hospitals where patients were referred for acute events.

### Participants and procedures

We considered eligible T2DM patients with age ≥40 years, persistent albuminuria ≥30 mg/24 h in at least two of three 24 h-urine collections in the last 6 months), severe DR (according to the Wilkinson et al.) [18], diabetes onset at age > 30 years, absence of neoplastic/psychiatric diseases and follow-up at the centre ≥ 12 months. Exclusion criteria were previous MI or stroke, severe hepatic or cardiac failure.

Patients were enrolled between October 2005 and October 2008. The intervention phase was scheduled for a period of four years, and it was completed in December 2011. Then, patients were followed until May 2019 to achieve the number of events needed for the primary outcome.

The protocol was approved by the ethics committee of University of Campania “Luigi Vanvitelli” (clinicaltrials.gov: NCT00535925) and is in accordance with the 1976 Declaration of Helsinki and its later amendments. All participants signed their informed consent.

### Randomization

All patients enrolled in each clinic were randomized, according to a cluster-randomization procedure, in two arms, MT and SoC. Randomization of centres was stratified based on their size, in order to reduce difference in the number of patients allocated to the two treatment arms.

The intensified therapy group was initiated to the therapeutic regimen summarized below and detailed and in Additional file 1: Appendix S1. Patients assigned to the conventional therapy group followed the therapy usually administered at their outpatient clinic; hence, they could receive any therapeutic change considered appropriate by their caregiver, under the respect of the good clinical practice rules.

### Targets

In either arm participating physicians were required to adhere to guideline-based clinical targets recommended at the time of study initiation: [11–14] (a) systolic blood pressure (SBP) < 130 mmHg, (b) diastolic blood pressure (DBP) < 80 mmHg, (c) glycated haemoglobin (HbA1c) < 7%, (d) fasting serum LDL cholesterol < 100 mg/dL, (e) fasting serum HDL cholesterol > 40/50 mg/dL (for men/women, respectively), and (f) fasting total serum cholesterol < 175 mg/dL.

### Study arms

In SoC group, the subjects received the therapy usually administered at their diabetic outpatient for the management of blood pressure, glycaemic and lipid control, and antiplatelet treatment. During the study, these patients received all therapeutic modifications considered appropriate by their physician, in the respect of the good clinical practice.

In MT group, the patients were treated with pre-specified algorithm for management of hypertension, glycol-metabolic control and dyslipidemia, including non-pharmacological and pharmacological treatment, as detailed in Additional file 1: Appendix 1. Briefly, specific recommendation for physical activity and low sodium diet were provided to patients in written form. In addition, renin–angiotensin system blockade was implemented by initial association of ACEi and ARBs with a strict monitoring of GFR and serum potassium, followed by stepwise addition of other anti-hypertensive drug classes. They received low-dose aspirin as primary prevention, unless contraindicated or not tolerated. Statin was added if non-pharmacological therapy was ineffective in reaching the target.

All patients, regardless of the study group, underwent control visits at their diabetes centre every six months to monitor laboratory and clinical parameters and compliance to therapies and lifestyle hints. During each visit, investigators carefully monitored the occurrence of adverse events. In MT group, additional visits could be planned if one or more risk factors resulted out of target. At each visit, adherence to pharmacological protocol as well as to lifestyle recommendations (see Additional file 1: Appendix S1) was strictly monitored and strengthened.

eGFR was estimated using the CKD-EPI equation and, since creatinine was not standardized, we reduced creatinine values by 5% [19].

### Outcomes

Primary endpoint was a composite of fatal and non-fatal MACEs, including cardiovascular mortality, non-fatal MI (documented instrumentally and/or enzymatically), non-fatal stroke, coronary-artery by-pass, revascularization procedures (PTCA) and lower limbs major amputation, whichever occurred first. In both arms, all endpoints were captured and recorded by investigators in an electronic Case Report Form (CRF) at each visit.

Since the planned number of events was not reached during the initial 4-year time frame (interventional phase), incidence of the primary end point was assessed throughout the follow-up phase, that in the original study design was planned to assess the durability of effects of the intensified treatment.

During this extension phase, following the end of intervention, all patients enrolled in both arms were treated by their own physicians according to the good clinical practice.

As secondary endpoints, we considered each single component of primary endpoint, and all-cause death at the end of the follow-up phase, as well as MACEs and the achievement of BP, HbA1c and total, HDL and LDL cholesterol goals at the end of intervention phase.

### Sample size

Study is powered to detect a Hazard Ratio (HR) of 0.67 in the comparison of the two groups, with an 80% power and a two-sided type I error of 5%, assuming an intraclass correlation coefficient of 0.01. For this purpose, with a sample size of about 420 patients, 14 overall clusters, an average of 30 subjects per cluster, and an expected surviving proportion of 30% at ten years in the SoC group, we determined a number of events needed of 258.

### Statistical analysis

All statistical analyses were performed after the end of the follow-up and the achievement of number of events needed for the analysis of the primary outcome. A statistical analysis plan was prepared before the central database was locked for final data extraction and analysis. Categorical data were expressed as number and percentage, while continuous variables as either median and interquartile range or mean and standard deviation, based on their distribution assessed by the Shapiro–Wilk test. In order to check for imbalance in cluster randomization, we compared variables at baseline by using the method proposed by Leyrat et al. [20] Standardized differences (SDiff) were calculated for continuous and dichotomous variable. P-values to take into account clustering were computed by generalized estimating equations (GEE) model with cluster as group variable [21]. Distribution of dependent variable and link function was used as appropriate (gaussian and identity for continuous variable, binomial and logit for dichotomous variable). Comparison of groups at end of intervention was performed applying the same methodology, further adjusting for baseline values as covariate.

Criteria on SDiff cut-offs reported by Leyrat et al. [20] were used to establish covariates imbalanced at baseline. Moreover, to evaluate a global imbalance, *c-statistic* was calculated performing a logistic model with treatment arm as dependent variable and selected baseline variables as covariate.

Median follow-up time was calculated by the inverse Kaplan–Meier procedure. The primary endpoint was analysed according to the intention-to-treat principle, with event curves for the time-to-first event based on Kaplan–Meier analysis. Cox regression model was used to calculate HR and 95% Confidence Interval (CI). Due to the cluster randomized study design, a Cox shared-frailty model was fitted. Across centres, the frailties are assumed to be gamma-distributed latent random effects affecting the hazard multiplicatively. In the univariate analysis, only treatment group was included as covariate. In the multivariable analyses, depending on imbalance detection of each variable (Leyrat method), association with the outcome of interest and evidence from the literature, we adjusted the Cox regression models for age, sex, SBP, haemoglobin, eGFR, albuminuria, HbA1c, total cholesterol, triglycerides (log-scaled), statins and antiplatelets therapy at baseline to reduce risk of bias. Data were analysed using STATA 16.0 software (StataCorp. 2019. College Station, TX: StataCorp LLC).

## Results

### Patients

**Fig. 1:**
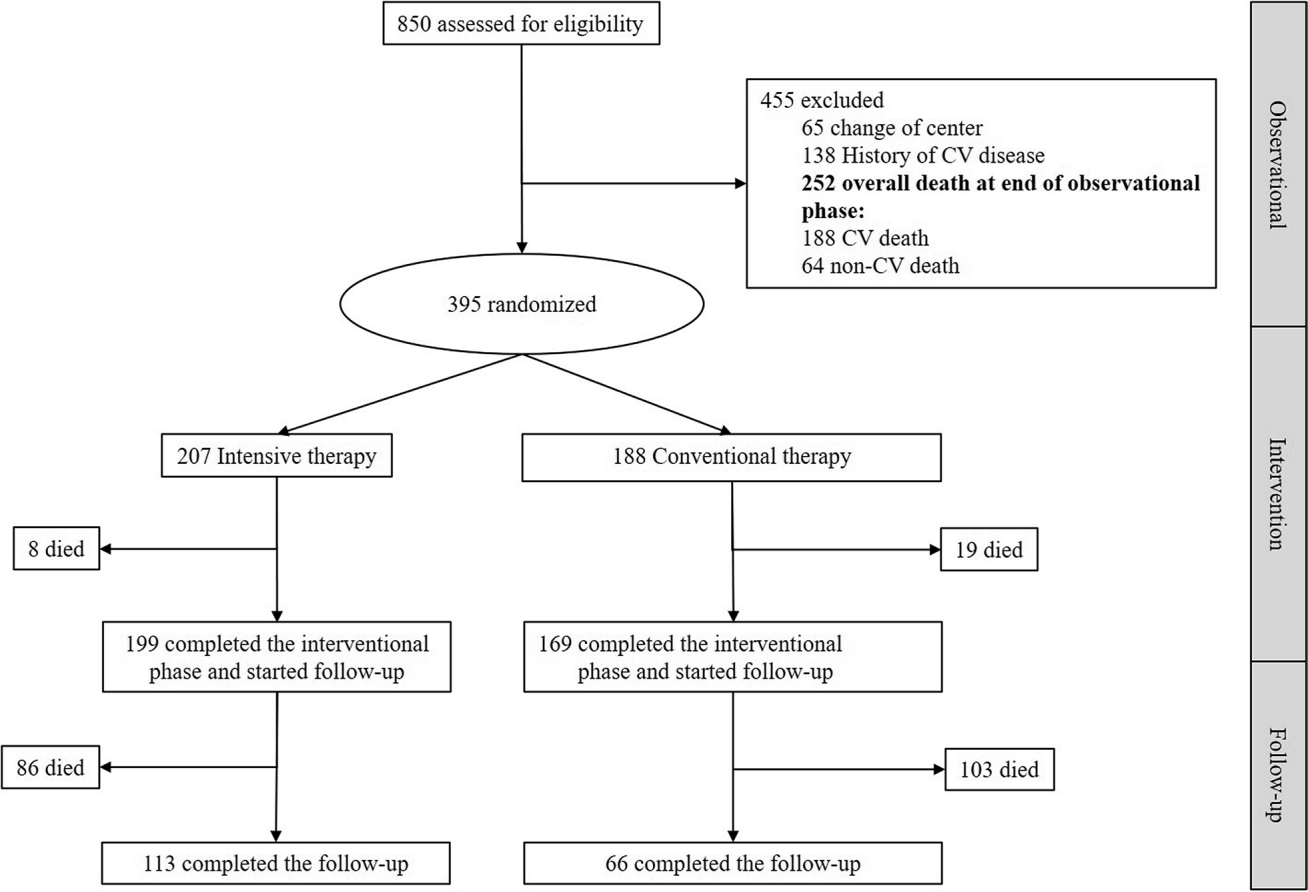
Flow chart of patients participating to the study

Out of the 850 eligible patients originally enrolled in the NID2 cross-sectional study [10, 22], we randomized 395 patients (207 to MT arm and 188 to SoC arm). Flow chart is reported in Fig. 1. Intervention and SoC groups were mostly similar for baseline characteristics (Table 1). However, patients in the group SoC were slightly older than those randomized to MT arm. HbA1c goal was more prevalent among controls. All variables, except for creatinine, showed a value of SDiff higher than 5%. These values are reported in Additional file 2: Fig. S1 to provide information on the direction (treatment arm) of the imbalance. Overall, SoC arm showed a more favourable baseline clinical picture compared with the intervention arm. Global imbalance, estimated using *c-statistic*, was around 0.8.

**Table 1:** Baseline characteristics of the study population (n = 395)

Parameter	SoC (n = 188)	Intervention (n = 207)	p	SDiff (%)
Male Sex, No. (%)	83(44.1)	103 (49.8)	0.382	11.3
Age, mean (SD), y	68.2 (8.8)	66.1 (9)	0.046	24.1
BMI (kg/m^2^), mean (SD)	29.4 (4.9)	28.5 (4.7)	0.288	19.1
Blood pressure (mmHg), mean (SD)				
Systolic	134.7 (12.6)	133.8 (14.3)	0.791	6.1
Diastolic	78.3 (7.7)	80.8 (7.4)	0.002	− 32.8
Systolic BP target, No. (%)	95 (50.5)	112 (54.1)	0.142	− 17.0
Diastolic BP target, No. (%)	150 (79.8)	136 (65.7)	0.274	19.2
Blood Pressure Target, No. (%)	90 (47.9)	105 (55.3)	0.193	− 14.8
Creatinine (mg/dL), mean (SD)	1.16 (0.5)	1.17 (0.5)	0.564	− 1.4
eGFR EPI-CKD (mL/min/m^2^), mean (SD)	62.7 (21.2)	65.4 (23)	0.350	− 12.1
eGFR EPI-CKD stage, No. (%)				
1	20 (10.6)	45 (21.7)	0.600	− 31.1
2	80 (42.6)	77 (37.2)		
3	76 (40.4)	71 (34.3)		
4	10 (5.3)	11 (5.3)		
5	2 (1.1)	3 (1.5)		
Albuminuria (mg/day), median [IQR]	57.3 [35—158.1]	120.5 [75.8 – 223.8]	0.115	− 25.2
Haemoglobin (mg/dL), mean (SD)	13.4 (1.3)	12.9 (1.5)	0.377	36.8
Glycemia (mg/dL), mean (SD)	152.7 (49)	155.9 (43.1)	0.924	− 6.9
HbA1c (%), mean (SD)	7.3 (1.1)	7.5 (1.1)	0.345	− 18.2
HbA1c Target, No. (%)	100(53.2)	66 (31.9)	0.001	43.1
Diabetes duration, median [IQR], y	9 (6–15)	9 (7–16)	0.801	− 11.4
Cholesterol (mg/dL), mean (SD)				
Total	185.8 (34.1)	187.6 (33.2)	0.688	− 5.5
LDL	111.5 (30.8)	107.5 (27)	0.703	13.7
Total Cholesterol Target, No. (%)	67 (35.6)	64 (30.9)	0.475	5.6
HDL Cholesterol Target, No. (%)	99 (52.7)	75 (36.2)	0.237	14.5
LDL Cholesterol Target, No. (%)	73 (38.8)	69 (33.3)	0.911	6.2
Triglycerides (mg/dL), median (IQR)	111 (88 – 153)	148 (115 – 190)	0.063	− 42.7
*Therapy*				
Anti-hypertensive Therapy, No. (%)			0.791	− 24.8
1	105 (55.9)	94 (45.4)		
2	20 (10.6)	31 (15)		
3	32 (17)	44 (21.3)		
4	18 (9.6)	27 (13)		
5	13 (6.9)	11 (5.3)		
ACEi/ARBs, No. (%)				
ACEi	119 (63.2)	125 (60.2)	0.748	− 25.6
ARBs	66 (35.4)	77 (37.4)		
ACE + ARBs	3 (1.4)	5 (2.4)		
Diuretics, No. (%)	83 (44.1)	113 (54.6)	0.840	− 21.0
Calcium Channel Blockers, No. (%)	63 (33.5)	82 (39.6)	0.792	− 12.7
Beta-blockers, No. (%)	31 (16.5)	38 (18.4)	0.655	− 4.9
Alpha-blockers, No. (%)	13 (6.9)	11 (5.3)	0.395	6.7
Diabetes Therapy, No. (%)			0.361	− 27.7
Diet	16 (8.5)	5 (2.4)		
Insulin	50 (26.6)	60 (29)		
Oral anti-hyperglycemics*	97 (51.6)	108 (52.2)		
Combined Therapy	25 (13.3)	33 (15.9)		
Missing	0 (–)	1 (0.5)		
Statins, No. (%)			0.569	− 10.8
Yes	72 (38.3)	89 (43)		
Missing	0 (–)	3 (1.4)		
Antiplatelets, No. (%)			0.057	− 58.8
Yes	71 (37.8)	126 (60.9)		
Missing	0 (–)	16 (7.7)		

p-values were computed by generalized estimating equations (GEE) model with cluster as group variable

*BMI *Body Mass Index, *BP* Blood Pressure, *GFR* Glomerular Filtrate, *HbA1c* Glycated haemoglobin, *IQR* interquartile range, *SD* standard deviation, *ACEi* Angiotensin-Converting-Enzyme inhibitor, *ARBs* Angiotensin II receptor blockers

*Metformin, Pioglitazone, acarbose, etc

### Intervention output

The median duration of intervention was 3.84 and 3.40 years in MT and SoC group, respectively. At the end of the intervention phase period, we found a significantly lower level of SBP, HbA1c, total and LDL cholesterol in the intensive-treatment arm (Table 2).

**Table 2:** Differences in demographic, clinical and laboratory parameters and pharmacological treatment between SoC and Intensive therapy group at end of intervention

Parameter	End of treatment		
	SoC Group (n = 169)	Intervention group (n = 199)	p**
Systolic blood pressure (mmHg)	135.1 (15.2)	127.3 (8.7)	0.004
Diastolic blood pressure (mmHg)	78.8 (8.8)	78.1 (5.6)	0.110
* BP* < *130/80 mmHg (%)*	132 (74.2)	163 (84.5)	0.045
*Laboratory tests*			
eGFR EPI-CKD (mL/min)	60.7 (22.9)	60.4 (22.5)	0.920
Albuminuria (mg/day)	90.2 (38–160)	54 (11–180)	0.179
* Albuminuria* < *30 mg/day (%)*	18 (13.0)	62 (37.6)	0.047
Fasting plasma glucose (mg/dL)	153.6 (44.8)	147.4 (39.4)	0.199
HbA1c (%)	7.4 (1.1)	6.9 (0.6)	0.009
* HbA1c* < *7% (%)*	88 (52.1)	129 (64.8)	0.133
Total cholesterol (mg/dL)	190.9 (33)	173 (28.9)	0.015
*Total cholesterol* < *175 mg/dL (%)*	55 (32.5)	106 (53.2)	0.054
LDL cholesterol (mg/dL)	122.3 (29.8)	100.5 (26.5)	< 0.001
*LDL* < *100 mg/dL (%)*	34 (20.1)	106 (53.2)	0.001
Triglycerides (mg/dL), median (IQR)	122 (90–171)	145 (120–169)	0.794
*Therapy*			
Anti-hypertensive drugs, median (IQR)	1 (1–3)	2 (1–3)	< 0.001
ACEi/ARBs, No. (%)			
* ACEi*	104 (61.8)	7 (3.4)	
* ARBs*	56 (33.1)	5 (2.3)	
* ACE* + *ARBs*	3 (1.8)	187 (94.3)	
* None*	6 (3.6)	–	< 0.001
Diuretics, No. (%)	84 (49.7)	113 (56.8)	0.864
Calcium channel Blockers, No. (%)	59 (34.9)	79 (39.7)	0.687
Beta-blockers, No. (%)	30 (17.8)	38 (19.1)	0.961
Alpha-blockers, No. (%)	6 (3.6)	10 (4.8)	0.574
Diabetes therapy, No. (%)			0.843
* Diet*	5 (2.9)	5 (2.5)	
* Insulin*	57 (33.7)	73 (36.7)	
* Oral anti-hyperglycemics*	77 (45.6)	88 (44.2)	
* Combined therapy*	16 (9.5)	25 (12.6)	
* Missing*	14 (8.3)	8 (4)	
Use of statins, No. (%)	84 (49.7)	110 (55.3)	0.991
Use of antiplatelets, No. (%)	104 (61.6)	148 (74.4)	0.905

Data are mean (SD) or median [IQR]

*P* Blood Pressure, *GFR* Glomerular Filtration rate, *HbA1c* glycated haemoglobin, *ACEi* Angiotensin-Converting-Enzyme inhibitor, *ARB* Angiotensin II receptor blockers

** P values refer to difference between SoC and Intensive therapy at the end of treatment and were computed by generalized estimating equations (GEE) model with cluster as group and adjusted with baseline value as covariate

**Fig. 2:**
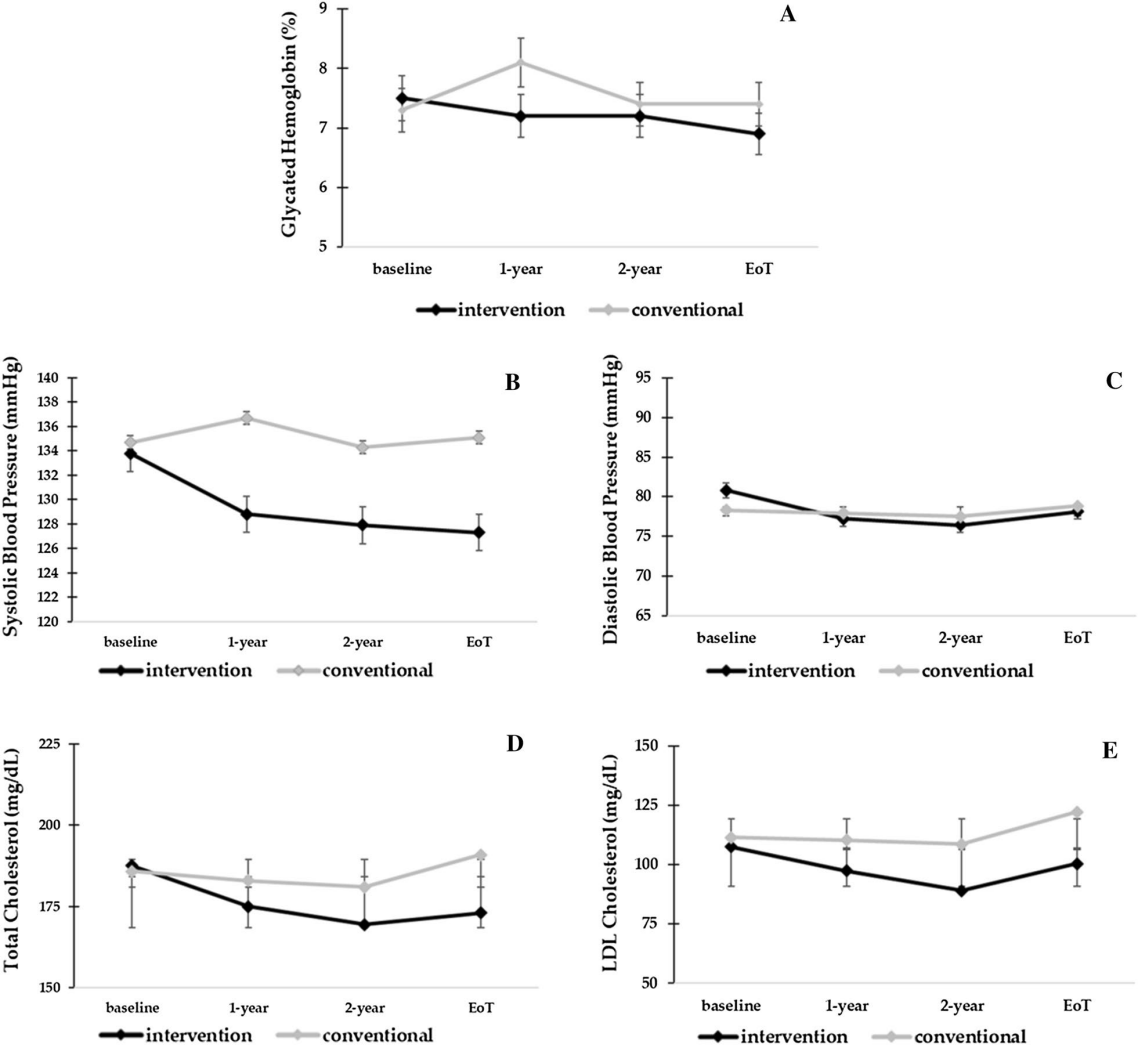
Changes in Blood Pressure, Glycemic and Lipids Control during the Interventional Study Period in the two arms of the study. **A**–**E** Mean (± SD) values for selected risk factors during the interventional part of the study for patients under Standard of Care (SoC) treatment (grey lines) and multifactorial therapy (MT) (black lines). Mean values were obtained at baseline, after 1-year, after 2-years and at end of interventional period (3.84 years in MT and 3.40 years in SoC). At these intervals, the total numbers of patients in both study groups were 395, 380, 372, and 368, respectively

In particular, during the Interventional Study Period, we observed a significant decline of BP, HbA1c and lipids in the MT arm already after the first year of intervention, after which mean values remained almost stable; conversely, no significant changes were detected in SoC group (Fig. 2).

In MT group, achievement of BP target < 130/80 mmHg was significantly higher overall (68.3% vs 50.9%, p < 0.001) and by component (SBP 77.4% vs 55.6%, p < 0.001 – DBP 81.9% vs 78.1%, *P* = *0.003*) (Additional file 3: Fig. S2). Similarly, HbA1c, total and LDL cholesterol targets were achieved more frequently in the intensive-treatment arm.

In MT arm, most of patients reached ≥ 4 targets (53% vs 29% in SoC arm); similarly, all six planned targets were more frequently achieved in the intervention arm (11.1% vs 2.4%).

Both hyperkaliemia (K^+^  > 5.5 mEq/L) and renal impairment (eGFR reduction > 30% than baseline values) were observed more frequently in the MT arm than in SoC arm [19 (10%) vs. 9 (5%); *P* = *0.120* and 30 (16%) vs 15 (9%), *P* = *0.080*, respectively), likely due to more frequent use of dual blockade of RAS with ARBs and ACEi association. Nonetheless, events leading to permanent ACEi/ARB or double block discontinuation were infrequent: hyperkaliemia (8; 4.1% in the MT group vs. 4; 2.1% in the SoC group; *P* = *0.558*) and renal impairment (3; 1.6% in the MT group vs. 2; 1.2% in the SoC group; *P* = *1.000*).

### Survival analysis

**Fig. 3:**
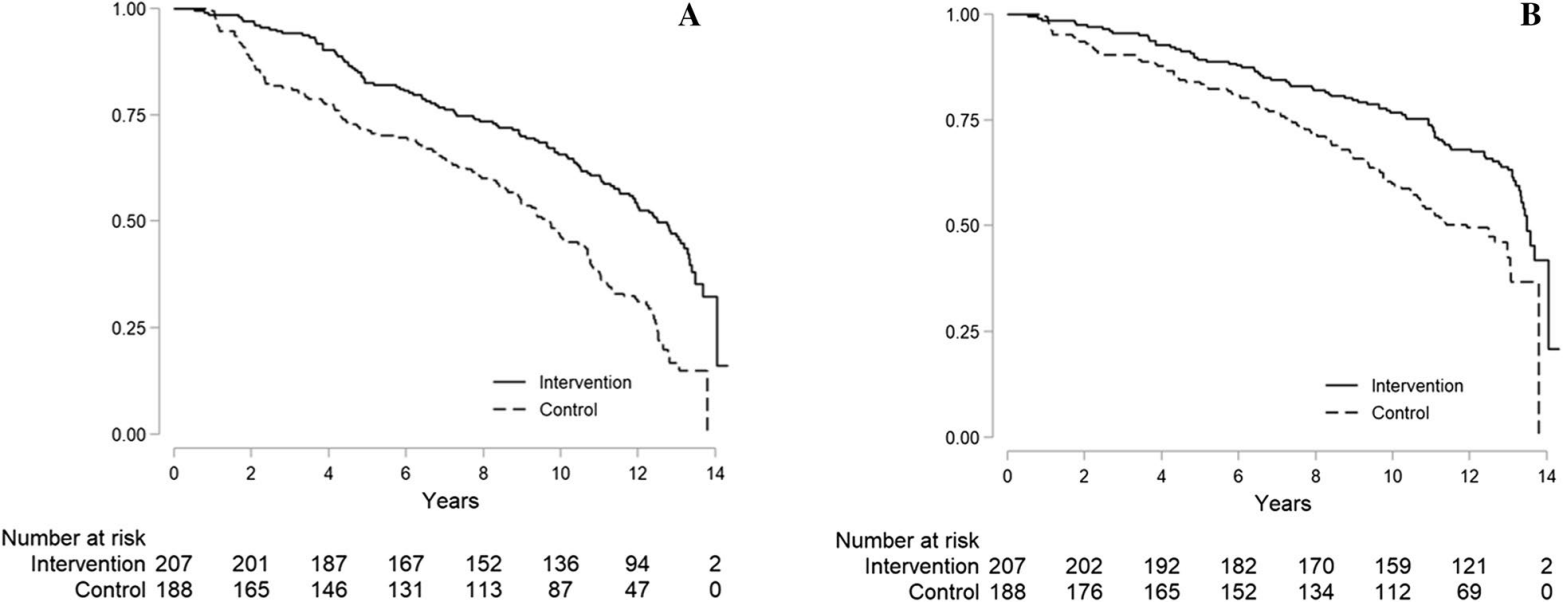
Kaplan–Meier Estimates of the Composite Endpoint of MACEs during the whole study period (intervention and follow-up) (**A**) and of mortality during the whole study period (intervention and follow-up) (**B**) in the Conventional-Therapy and Intensive-Therapy Groups, respectively

During follow-up (median 13.0 years, IQR 12.4–13.3). 262 MACEs were recorded, 146 in the SoC group and 116 in the intensive-therapy group (Table 3). Kaplan Meier analysis (Fig. 3A) showed a major difference between the two arms, with a median survival of 9.6 years (95% CI 8.6–10.7) for SoC group and 12.5 years (95% CI 11.5–13.3) for intensive-therapy group. The Cox shared-frailty model confirmed this finding at univariate analysis (HR 0.49, 95% CI 0.35–0.69, *P* < 0.001), as after multiple adjustments (adjusted HR 0.47, 95% CI 0.30–0.74, *P* = *0.001*).

**Table 3:** Clinical outcome in the two groups (n = 395)

Event	SoC group (n = 188)	Intervention group (n = 207)	p*
End of follow-up			
MACEs, No. (%)	146 (77.8)	116 (56.1)	< 0.001
All-cause death, No. (%)	103 (54.8)	86 (41.6)	0.046
Myocardial infarction, No. (%)	44 (23.4)	32 (15.5)	< 0.001
Stroke, No. (%)	28 (14.9)	19 (9.2)	0.002
Revascularization, No. (%)	13 (6.9)	16 (7.8)	0.33
Major amputation, No. (%)	3 (1.6)	2 (0.9)	0.5
Follow-up at end of intervention phase			
MACEs, No. (%)	50 (26.6)	24 (11.6)	0.002

*MACE *Major Adverse Cardiovascular Event

* p-value calculated with univariable Cox shared-frailty model. Multivariable estimates are reported in the manuscript

Secondary endpoints are reported in Table 3. All-cause mortality occurred in 189 patients (103 in the SoC group and 86 in the intensive-therapy group). The Kaplan–Meier analysis disclosed a median survival time of 11.9 years (95% CI 10.5–13.05) for SoC group and 13.5 years (95% CI 13.3–15.0) for intensive-therapy group (Fig. 3 panel B). The Cox shared-frailty model showed a significant difference both at univariate model (HR 0.58, 95% CI 0.34–0.98, *P* = *0.046*), and after adjustment for confounding variables (adjusted HR 0.53, 95% CI 0.29–0.93, *P* = *0.027*). Incidence of MI and stroke was lower in intensive-therapy group, while PTCA and major amputation did not differ. These inter-group comparisons were unadjusted because of the small number of events.

As additional secondary endpoint, we also analysed MACEs at the end of intervention phase. Overall, 74 MACEs were recorded, 50 in the SoC group and 24 in the intensive-therapy group (Table 3) with an unadjusted HR 0.28 (95% CI 0.13–0.63; *P* = *0.002*). During intervention phase, we recorded kidney failure (either initiation of chronic dialysis or eGFR < 15 mL/min/1.73 m^2^) in 12 patients (5 in the SoC group and 7 in the intensive-therapy group).

## Discussion

This cluster-randomized trial demonstrates that in DKD patients at very high cardiovascular risk, a multifactorial intensive treatment significantly reduces the risk of MACEs versus SoC. This cardiovascular benefit becomes evident early during the three years of intervention and persisted over the long-term follow-up of 13 years. Noteworthy, the favourable effect extends to all-cause mortality.

Several studies have demonstrated the high cardiovascular risk in T2DM. Among these, the large meta-analysis by the Emerging Risk Factors Collaboration centre showed a significantly higher adjusted risk among individuals with diabetes at younger ages and women, as well as a strong association with cardiovascular deaths [23]. Likewise, in the Swedish National Diabetes Register, a close association between CV events and the number of risk factors at goal was further disclosed [24]. This high residual risk is matter of debate. It may be possibly due to inadequate global management of risk factors. Indeed, the European Euro Heart Survey showed how a poly-pharmacological approach improved prognosis of patients with diabetes [25]. Nevertheless, a multi-drug therapy in T2DM does not guarantee for the gain of the different goals suggested by guidelines, as reported by the EUROASPIRE IV survey [26]. Such a gap between goals and real-life findings suggests the need of a multidisciplinary approach in diabetology outpatient clinics [27–30].

Although several observational studies have confirmed the association between risk factors and cardiovascular outcome, solid evidence on the efficacy of a multifactorial intervention in reducing the risk in DKD patients is limited. Indeed, only one randomized trial (Steno-2) has addressed this issue.[7, 8]. Steno-2 trial randomized 160 T2DM albuminuric patients either to an intensive multifactorial therapy or SoC. Although main therapeutic goals (HbA1c, lipids and BP) were not fully achieved in the intensive-treatment group, after a mean follow-up of 7.8 years, patients intensively treated had a significant 50% lower risk of both cardiovascular and microvascular events [7].

To date, the findings of Steno-2 study were not confirmed by other studies, even in those enrolling diabetic subjects regardless of DKD selection criterion. In multicentre, open-label, randomized, J-DOIT3 study [31], type 2 diabetes patients, not specifically selected with DKD, were randomly assigned to either multifactorial intensive or conventional therapy and followed up for a median of 8.5 years. Notably, the study did not evidence any efficacy of intensified multifactorial intervention compared with current standard care on the prevention of a composite of coronary events, cerebrovascular events, and all-cause mortality.

Of note, the present trial was originally conceived to overcome the limits of the Steno-2, in terms of study planning (we adopted a multicentre study design) as well as patients’ selection. Indeed, our population was older (67 vs 55 yrs), well balanced for sex (in Steno-2 men accounted for 75% study population) and with a lower eGFR (64 vs 117 ml/min/1.73 m^2^). These features make our population much closer to the albuminuric T2DM subjects from National Registries, and thus more representative of real-life population with diabetes [24, 32]. We originally enrolled only patients at primary cardiovascular prevention, unlike Steno-2, which also recruited patients with prior cardiovascular events. Furthermore, our cohort had DR as inclusion criterion. The combination of albuminuria and DR portends a higher cardiovascular risk [33]. The higher cardiovascular risk in our study population vs Steno-2 cohort more likely explains the effectiveness of a shorter multifactorial treatment (3.84 vs. 7.8 years in Steno-2). Moreover, in our study, the risk reduction induced by intensive treatment occurred earlier than in Steno-2; indeed, frailty model showed a significantly lower risk for overall mortality and MACEs, already evident at end of intervention phase.

The pathophysiological mechanisms underlying the cardiovascular protective effect in MT arm can only be hypothesized. Besides the well-known effects of BP lowering treatment with RAS inhibition, the intensive glycaemic control with the consequent “legacy effect” on AGEs [33], the effect of statin on LDL and inflammatory cytokines [34, 35], and the inhibition of platelet adhesion, which reduces leukocyte infiltration and atherosclerosis [36], more likely, the combination of different interventions may have been critical.

In our study, centres allocated to intensified therapy achieved a higher rate of patients at target for both single and multiple risk factors. Of note, the intervention study took place before GLP1-RA and SGLT2i had been marketed in Italy. Therefore, these drugs, though characterized by important cardioprotective effect [1], could not be assessed.

In the NID-2 study, the intervention phase was completed in 2011, prior to the publication of the Veterans Affairs Nephropathy in Diabetes (VA-NEPHRON-D) study, [37] which firstly showed in diabetic nephropathic patients an association between the combination of ACEi and ARB and an increased risk either of decreased eGFR and/or of the onset of hyperkaliaemia. However, the NID-2 protocol required a strict monitoring of renal function and serum electrolytes and required the permanent withdrawal of dual RAS blockade in the case of persistent side effects, as above reported. Notably, during intervention phase the percentage of subjects who had to suspend treatment with drugs active on the RAS due to persistent side effects was low and was similar to what was recorded in the Altitude study [38].

Our study has several strengths. First, it is the first multicentre study showing the effectiveness of a multifactorial treatment on MACEs. Second, durability of our findings was confirmed by means of a long follow-up. In addition, randomization by centre makes the study closer to real-life clinical practice. On this regard, it is noteworthy that the SoC arm showed a more favourable clinical picture at baseline in comparison with the intervention arm. This finding reasonably excludes the possibility of selecting in SoC arm those physicians with lower attitude to adhere to clinical guidelines. However, the cluster-randomized design has a number of limitations, such as the lack of blind assignment, power and precision are lower than an individually randomized trial and the ability to control for both known and unknown confounder is reduced. There is extensive discussion in the Literature on strategies and methods to reduce these limitations and mitigate the impact on results. We attempted at the best use of them to achieve internal validity so that unbiased estimates of efficacy can be obtained within the study population and generalized to target population [39]. Specifically, the main results of the study were adjusted for both the cluster factor due to randomization and for the main variables resulted either unbalanced or clinically and statistically associated with outcome.

As major limitation, we did not collect clinical and laboratory data during the follow-up occurring after intervention phase but only events of interest (death and cardiovascular events) thus precluding the possibility of identify a prevailing factor definitely associated with risk reduction. In addition, some factors possibly related to CV outcome, such as uric acid or proBNP [40, 41], were not included in our laboratory panel. Therefore, we cannot identify one or more specific factor definitely associated with risk reduction; however, our original aim was to test efficacy of a global approach rather than effects of single interventions.

Moreover, the choice of a composite of fatal and non-fatal MACEs as primary endpoint may explain the lack of a sufficient power for mortality outcome. Finally, the SoC population was two-years older than the intensified treatment one. However, considering the mean age in both groups > 65 years, the high CV risk as inclusion criterion, and both a higher mean DBP and a much lower prevalence of HbA1c target subjects at baseline in the intervention group, we believe the two groups are clinically comparable.

## Conclusions

Therefore, this multicentre trial demonstrates that a strategy based on intensive treatment of main risk factors is more effective than standard of care in preventing MACEs in type 2 DKD. Pursuing such a strategy triggers a marked improvement of outcomes occurring early after its implementation and lasting in the long-term.

## Supplementary Information

**Additional file 1: Appendix S1.** Intervention by randomization.

**Additional file 2: Figure S1.** Standardized Differences for Imbalance Detection. The standardized differences are reported on x- axis. The type of point describes the imbalance in favor of the treatment arm. The right panel shows the only variables for which a therapeutic achievement target has been calculated. Covariance Imbalance Detection: if SDiff was either ≥5% or <5% but its correlation with at least one covariate with a SDiff ≥5 was greater or equal to 0.2.

**Additional file 3: Figure S2.** Targets Achievement at the end of intervention phase. Type (left panel) and number (right panel) of target for single cardiovascular risk factors achieved at the end of intervention period in conventional therapy (black bars) vs Intensive therapy (gray bars).

## Data Availability

All data generated or analyzed during this study are included in this published article and its supplementary information files.

## Abbreviations

ACEi: Angiotensin converting enzyme inhibitor
ARBs: Angiotensin II receptor blockers
CRF: Case Report Form
DKD: Diabetic kidney disease
DR: Diabetic retinopathy
DBP: Diastolic blood pressure
GEE: Generalized estimating equations
HbA1c: Glycated haemoglobin
HR: Hazard Ratio
MACEs: Major fatal and non-fatal cardiovascular events
MT: Multifactorial intensive therapy
MI: Myocardial infarction
NID-2: Nephropathy in diabetes type 2
RAS: Renin-angiotensin system
PTCA: Revascularization procedures
SDiff: Standardized differences
SoC: Standard-of-Care
SBP: Systolic blood pressure
T2DM: Type 2 diabetes mellitus
