# Supplementary material for: Efficacy and durability of multifactorial intervention on mortality and MACEs: a randomized clinical trial in type-2 diabetic kidney disease
Source: Cardiovasc Diabetol. 2021 Jul 16;20:145. doi: 10.1186/s12933-021-01343-1 (PMC8285851; doi:10.1186/s12933-021-01343-1)
Supplement: Supplementary file 1 — Additional file 1: Appendix S1. Intervention by randomization. [file 12933_2021_1343_MOESM1_ESM.docx]

***Appendix S1 –Intervention by Randomization***

In SoC group, all enrolled patients were advised to follow the implementation of the rules of a correct lifestyle including smoking cessation, physical activity, weight loss, nutritional counseling. No specific pharmacologic algorithm were provided and physicians were left free of modifying therapy (type of drug and dosage) in order to achieve guideline-based clinical targets (systolic/diastolic blood pressure <130/80 mmHg, glycated haemoglobin <7%, fasting serum total, LDL and HDL cholesterol <175 mg/dL, <100 mg/dL and >40 in men and >50 mg/dL in women, respectively).

In MT group, the same targets were pursued; however, at variance with SoC, specific indications were provided to participating physicians. In particular, patients were asked to do at least 5 times a week 30 minutes of aerobic physical activity (e.g., walking, cycling,…) and try to keep a diary of this activity. In overweight or obese patients, a personalized diet, adequately illustrated, was further prescribed with specific advice of reducing their salt intake. This was pursued by the following recommendations: (1) remove the salt-shaker from the table; (2) consume unsalted bread; cooking without salt (i.e., add it only in smaller quantities after cooking); (3) use spices, lemon, vinegar, chili in place of salt, ketchup, mayonnaise, mustard; (4) get used to reading the sodium content on the labels of packaged foods; (5) eliminate salty snacks, cured meats and aged cheeses; (6) give preference to fresh foods over canned ones.

For **blood pressure management,** all patients assigned to the intervention subgroup were treated with dual blockade of RAS with an ACE inhibitor and an angiotensin receptor blocker (ARB) administered at the maximum tolerated dose (recommended: 300 mg irbesartan plus 5 mg ramipril, or equivalent) unless either intolerance or side effects (persistent cough, hypotension, hyperkalemia ≥6.0 mEq/L or increased creatinine >30% during the first 14 days from start of therapy). At each time-visit, if BP was not at goal, investigators were asked to increase the dose of BP drug or to add a new drug with two-week intervals until the achievement of the goal. The therapeutic flow-chart provides for the following **sequential** intervention scheme (if creatinine >2 mg/dL nephrological advice was required): (i) **Step 1** 🡪 ARB + Ace inhibitor (to the entire study population) (up to 300 mg irbesartan plus 5 mg ramipril); (ii) **Step 2** 🡪 Thiazide diuretic (added to drugs of Step1, 12.5-25 mg/day hydrochlorothiazide if creatinine ≤2 mg/dL, otherwise furosemide 25-75 mg/day); (iii) **Step 3** 🡪 Dihydropyridine calcium channel blocker; (iv) **Step 4** 🡪 Beta-blocker (atenolol up to 100 mg/day); (v) **Step 5** 🡪 Alpha-blocker; (vi) **Step 6** 🡪 Clonidine.

Steps 3, 4, 5 and 6 were interchangeable with each other, in the presence of compelling indications.

The **glyco-metabolic** control was assessed by HbA1c evaluation. Daily blood glucose profiles, to be performed at least every 15 days, served to optimize therapy. To achieve and maintain a good glycemic control in the intervention arm, the implementation of lifestyle tips (diet and physical activity), according to ADA recommendations was both stressed and periodically monitored at each visit by a personal diary.^10^

A handy and safe oral anti-hyperglycemic therapy (OHA) was used in nephropathic patients. In the case of GFR (CKD-EPI) <30 ml/min/1.73m^2^ metformin and sulfonylureas with renal excretion had to be avoided. In case of unsatisfactory control with either OHA or an insulin therapy scheme already in place, in the first case a switch to an add-on therapy was introduced (OHA + basal insulin) and, in the second, to an insulin scheme optimized basal bolus. If HbA1c was between 7% and 8%, it was monitored again within 2 months and, if confirmed, therapy was modified. If HbA1c >8% the change of therapy was immediate. In addition to the controls by protocol, HbA1c control will be repeated two to three months after each change of the therapy.

For **lipid control**, in case of failure to reach the targets, only three months of implementation of lifestyle advice were allowed. In the case of persistence of LDL cholesterol >100 mg/dL, the life-style modification was integrated by pharmacological therapy; in particular, a statin at adequate dosage to gain the target was introduced.

Low-dose aspirin (100 mg/day) was administered for primary prevention in all patients, unless contraindicated or not tolerated.
