# Supplementary figures and images for: Efficacy and durability of multifactorial intervention on mortality and MACEs: a randomized clinical trial in type-2 diabetic kidney disease
Source: Cardiovasc Diabetol. 2021 Jul 16;20:145. doi: 10.1186/s12933-021-01343-1 (PMC8285851; doi:10.1186/s12933-021-01343-1)

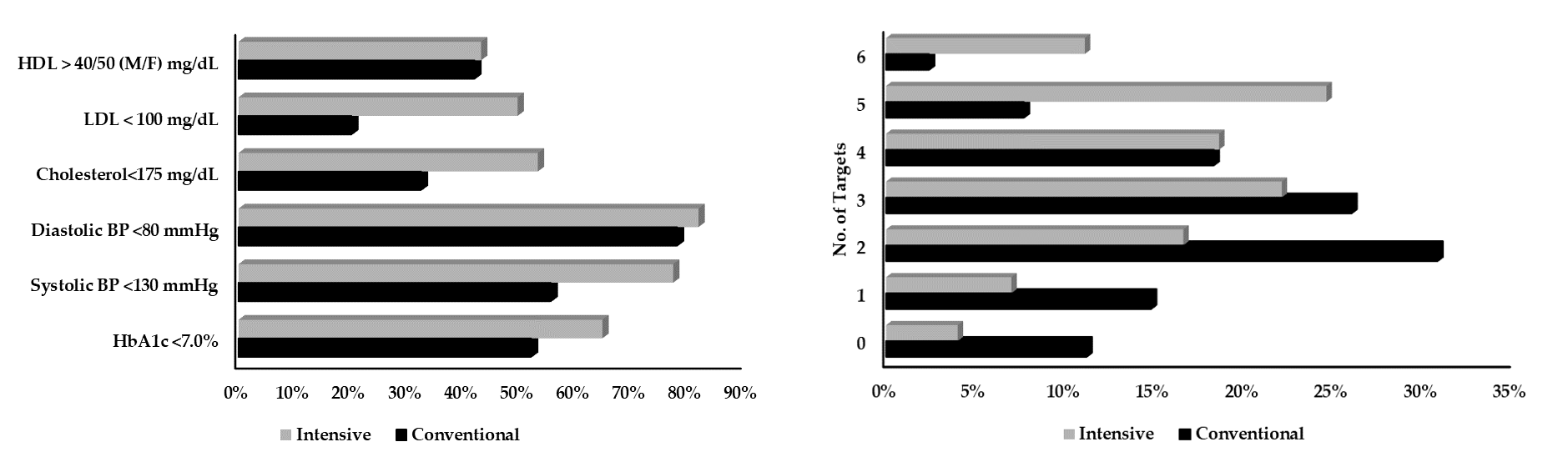

Supplement: Supplementary file 3 — Additional file 3: Figure S2. Targets Achievement at the end of intervention phase. Type (left panel) and number (right panel) of target for single cardiovascular risk factors achieved at the end of intervention period in conventional therapy (black bars) vs Intensive therapy (gray bars). [file 12933_2021_1343_MOESM3_ESM.tif]
